# Efficacy of ultra-early rehabilitation on elbow function after Slongo’s external fixation for supracondylar humeral fractures in older children and adolescents

**DOI:** 10.1186/s13018-022-03120-6

**Published:** 2022-04-07

**Authors:** Andreas Rehm, Azeem Thahir, Albert Ngu, Luke Granger

**Affiliations:** 1 Consultant Paediatric Orthopaedic Surgeon, Paediatric Division, Department of Paediatric Orthopaedics, Cambridge University Hospitals NHS Trust, Hills Road, Cambridge, CB2 0QQ UK; 2 Clinical Fellow, Department of Trauma & Orthopaedics, Cambridge University Hospitals NHS Trust, Cambridge, UK; 3 Higher Orthopaedic Trainee, Department of Trauma & Orthopaedics, Cambridge University Hospitals NHS Trust, Cambridge, UK; 4 Higher Orthopaedic Trainee, Paediatric Division, Department of Paediatric Orthopaedics, Cambridge University Hospitals NHS Trust, Cambridge, UK

**Keywords:** Supracondylar fracture, Supracondylar humerus fracture, Supracondylar humeral fracture, External fixator, External fixation, Slongo’s external fixator, Slongo external fixator, Rehabilitation, Elbow function, Paediatric elbow trauma, Pediatric elbow trauma, Flynn criteria

We read with interest the recent publication by He and colleagues [1]. The authors [1] stated that the time to regain the range of movement required for functional activity of daily living (ROM-ADL: flexion = 30°–130°; forearm rotation = 100° (50° pronation, 50° supination)) [2] was significantly shorter for the rehabilitation group (R-group) than the control group (C-group), but this is contradicted by the provided data listed in the authors’ first table (time to ROM-ADL: C-group = 6.65 ± 0.8 weeks; R-group = 9.74 ± 1.25 weeks). In our experience, it would be highly unlikely for a patient group who achieved ROM-ADL (poor Flynn score) at a mean of 9.74 weeks to then progress within 2.26 weeks to the high number of good and excellent Flynn scores as recorded by He et al. [1] at the 12 weeks assessment for the C- and R-group.

He et al. [1] stated that there was no significant difference in weight and height between the two groups, not identifying the huge difference in body mass index (BMI), with the C-group having a normal mean BMI of 22.4 and the R-group being obese with a mean BMI of 30.3. Golden et al. [3] reported a correlation between increased BMI and reduced range of movement (ROM) of the elbow, with an expected loss of ROM of about 11° to 17° for obese children because of a soft tissue block to full flexion. Therefore, He et al. [1] should have measured reduced flexion and total ROM of the un-injured arm for the obese R-group compared to the C-group but reported equal mean values instead, which would be highly unlikely considering Golden et al.’s data [3].

He et al. [1] did not describe how ROM was measured and did not test intra- and inter-observer reliability. The latter authors [1] reported a mean increase of the carrying angle between the 3 and 6 months assessments of 3.24° (66%) for the C-group and 3.42° (52%) for the R-group, which can only be a systematic measuring error, since such a change would not occur as a result of rehabilitation because it is a fixed angle and potential remodelling of a coronal deformity would take much longer than 3 months, if it occurs at all. Therefore, the reported differences for total elbow ROM and flexion between the C- and R-group at 3 months (3.01°/1.74°) and 6 months (3.36°/2.09°) are most likely not real differences but measuring errors.

He et al. [4] previously presented a comparison between a K-wire and an external fixator group, not mentioning a rehabilitation program for either group. He et al. [1, 4] reported exactly the same data (means and standard deviations) in both publications for age, weight, height, admission to surgery time and length of hospital stay for the K-wire group [4] and C-group [1] and external fixator group [4] and R-group [1], respectively, despite the K-wire- and C-group and the R- and external fixator group containing different numbers of patients. Such an exact match of multiple data is only possible, if both publications include data from the same groups, which would mean that the K-wire [4] and external fixator control group [1] data are from the same group (the same applies to the external fixator group [4] and rehabilitation external fixator group [1]), indicating that there has been an extensive data transcription error, which would invalidate the results and conclusions of the paper.

He et al. [1] provided intra-operative post-fixation radiographs show rotational fracture malalignment with displacement of the medial column as seen in the authors’ [1] images G and H. He et al. [4] previously provided radiographs and clinical photographs of a different external fixation child, showing a large radiographic extension deformity after fixation with marked loss of elbow flexion, loss of extension and loss of normal carrying angle, which we would grade as a poor outcome based on the Flynn criteria [5], but the authors [4] did not recognize it as such.

Slongo et al. [6] promoted their new technique of external fixation in 2008, stating that their method is very simple to use and can overcome the problem of achieving an unsatisfactory reduction, as seen with Kirschner wire (K-wire) fixation. In contrary to this, the only provided lateral radiograph taken following fixation shows a mal-reduced fracture with a large extension deformity and possible malrotation. Slongo [7] represented his new technique in 2014, when he described the radiographic position of the same mal-reduced fracture as “perfect”, with all included post-fixation lateral radiographs of other patients showing various degrees of mal-reductions, with extension and/or rotational deformities. Despite the latter, the author [7] described poor elbow flexion (Flynn score) of about 115° as seen on the publication’s last clinical photograph as “full function”.

In conclusion, the presented radiographic and photographic clinical evidence provided by He et al. [1, 4] and Slongo et al. [6, 7] indicates to us that Slongo’s external fixation technique is not as simple as it was described, with there being a mismatch between the presented radiographic mal-reductions and poor photographic clinical findings and the reported good outcome data, which does not support that the use of external fixation for the management of supracondylar humerus fractures is easier and superior compared to K-wire fixations. The data do also not support that He et al.’s [1] very labour-intensive ultra-early rehabilitation results in better outcomes compared to self-rehabilitation.

## Data Availability

Not applicable.

## Abbreviations

BMI: Body mass index
C-group: Control group
R-group: Rehabilitation group
ROM: Range of movement
ROM-ADL: Range of movement required for functional activity of daily living
